# An Evaluation of the Service Intensity Add-On Payment Policy Reform in the Medicare Hospice Benefit

**DOI:** 10.1093/geroni/igab046.237

**Published:** 2021-12-17

**Authors:** Thomas Christian, Pedro Gozalo, Joan Teno, Michael Plotzke

**Affiliations:** 1 Abt Associates, Cambridge, Massachusetts, United States; 2 Brown University, Providence, Rhode Island, United States; 3 Oregon Health and Science University, Portland, Oregon, United States; 4 Abt Associates, Saint Louis, Missouri, United States

## Abstract

In 2016, the Centers for Medicare & Medicaid Services (CMS) implemented the Service Intensity Add-On (SIA) payment, which incentivized skilled nurse and medical social worker (SN/MSW) visits in the last seven days of life. Little is known about the impact of this initiative. Using 100% Medicare hospice claims, we identified a 10% random sample of Medicare hospice beneficiaries utilizing routine home care service during calendar years 2012-2018. We compared the provision of SN/MSW visits on service dates before and after the SIA’s implementation relative to beneficiaries’ date of death. We also determined hospice providers’ success in providing SN/MSW visits in the last days of life and categorized all providers into quintiles according to the average rate of these visits in the period prior to the SIA’s implementation. Cumulative over the last seven days of life, we calculated an increase of 15.7 SN/MSW minutes (95% confidence internal [CI] 14.9-16.5 minutes) per beneficiary after the SIA was implemented. The per-minute increase was greatest on days nearer to death (4.0 minutes day of death, 95% CI 3.6-4.2). There was no detectable visit increase on days which were ineligible for the SIA. Additionally, those providers in the quintile providing the lowest rate of SN/MSW visits pre-SIA exhibited a 14-percentage point increase in rates of these visits, the third, fourth, and fifth quintiles exhibited little change over time. Further monitoring is needed to ensure beneficiaries receive adequate end-of-life care.

